# Acute- and late-phase matrix metalloproteinase (MMP)-9 activity is comparable in female and male rats after peripheral nerve injury

**DOI:** 10.1186/s12974-018-1123-7

**Published:** 2018-03-20

**Authors:** Albert G. Remacle, Swathi K. Hullugundi, Jennifer Dolkas, Mila Angert, Andrei V. Chernov, Alex Y. Strongin, Veronica I. Shubayev

**Affiliations:** 1 Infectious and Inflammatory Disease Center/Cancer Research Center, Sanford Burnham Prebys Medical Discovery Institute, La Jolla, San Diego, CA 92037 USA; 2 Department of Anesthesiology, University of California, 9500 Gilman Drive, La Jolla, San Diego, CA 92093-0629 USA; 3 VA San Diego Healthcare System, La Jolla, San Diego, CA 92037 USA

## Abstract

**Background:**

In the peripheral nerve, pro-inflammatory matrix metalloproteinase (MMP)-9 performs essential functions in the acute response to injury. Whether MMP-9 activity contributes to late-phase injury or whether MMP-9 expression or activity after nerve injury is sexually dimorphic remains unknown.

**Methods:**

Patterns of MMP-9 expression, activity and excretion were assessed in a model of painful peripheral neuropathy, sciatic nerve chronic constriction injury (CCI), in female and male rats. Real-time Taqman RT-PCR for MMP-9 and its endogenous inhibitor, tissue inhibitor of metalloproteinase-1 (TIMP-1) of nerve samples over a 2-month time course of CCI was followed by gelatin zymography of crude nerve extracts and purified MMP-9 from the extracts using gelatin Sepharose-beads. MMP excretion was determined using protease activity assay of urine in female and male rats with CCI.

**Results:**

The initial upsurge in nerve MMP-9 expression at day 1 post-CCI was superseded more than 100-fold at day 28 post-CCI. The high level of MMP-9 expression in late-phase nerve injury was accompanied by the reduction in TIMP-1 level. The absence of MMP-9 in the normal nerve and the presence of multiple MMP-9 species (the proenzyme, mature enzyme, homodimers, and heterodimers) was observed at day 1 and day 28 post-CCI. The MMP-9 proenzyme and mature enzyme species dominated in the early- and late-phase nerve injury, consistent with the high and low level of TIMP-1 expression, respectively. The elevated nerve MMP-9 levels corresponded to the elevated urinary MMP excretion post-CCI. All of these findings were comparable in female and male rodents.

**Conclusion:**

The present study offers the first evidence for the excessive, uninhibited proteolytic MMP-9 activity during late-phase painful peripheral neuropathy and suggests that the pattern of MMP-9 expression, activity, and excretion after peripheral nerve injury is universal in both sexes.

## Background

Peripheral neuropathy arises from lesion and disease affecting the peripheral nervous system. There are over 100 peripheral neuropathy types associated with metabolic disease, nutritional deficiencies, trauma and exposure to drugs, toxins, alcohol, and viral pathogens, with neurological symptoms including muscle weakness, numbness, loss of autonomic functions, and debilitating neuropathic pain [1]. The mechanisms underlying pathological changes in peripheral neuropathy depend on the triggers and may be influenced by biological variables, including sex and gender. Recent experimental evidence highlighted distinct immune response mechanisms to peripheral nerve injury in the development of mechanical hypersensitivity in female and male rodents [2]. These recent data added to the body of evidence of sexual dimorphism in the formation of collateral axonal sprouting, cortical connectivity, and the activities of brain-derived neurotrophic factor, *N*-methyl-D-aspartate, and opioid receptors in the damaged nervous system (reviewed in [3]).

The matrix metalloproteinase (MMP) family of calcium-dependent zinc endopeptidases includes soluble (collagenases, gelatinases, matrilysins, and stromelysins) and membrane-type MMPs [4]. The structure of soluble MMP comprises an N-terminal inhibitory prodomain followed by an active site catalytic domain, a flexible linker region, and a C-terminal hemopexin domain. MMPs are synthesized as latent inactive proenzymes that require the proteolytic removal of their inhibitory prodomain to expose the active site and generate the catalytically active mature proteases. MMP proteolysis regulates the levels and the functionality of extracellular matrix components and cell surface signaling receptors [5]. In the damaged adult peripheral nerve, pro-inflammatory MMP-9 (gelatinase B) is produced uniquely and immediately after injury by Schwann, endothelial, and immune cells to regulate the blood-nerve barrier breakdown, immune cell recruitment, glial activation, demyelination, remyelination, and pain [6–16].

MMP activity is regulated by four tissue inhibitors of MMPs (TIMPs), each comprised of the N-terminal inhibitory and the C-terminal non-inhibitory domains. Among TIMPs, TIMP-1 is the most efficient inhibitor of the pro-inflammatory MMP-9 gelatinase [4]. In addition, TIMP-1 via its C-terminal domain forms a unique stoichiometric (1:1), stable heterodimer complex with the hemopexin domain of MMP-9 proenzyme. This complex is significantly more resistant to activation relative to the TIMP-1-free MMP-9 proenzyme. Because TIMP-1 is highly expressed in the naïve rat nerve [17, 18] and is further upregulated concomitantly to MMP-9 in the acute phase of nerve injury [17, 18], MMP-9 is found predominantly as a latent inactive proenzyme [7, 8, 17, 18].

Whether MMP-9 is expressed and active in late-phase painful peripheral nerve injury remains unknown. In light of the emerging evidence of sexual dimorphism in neuroimmune pathogenesis of neuropathic pain progression after peripheral nerve injury [2], we here aimed to determine the patterns of MMP-9 expression, activity, and excretion utilizing a widely used model of painful peripheral neuropathy, rat sciatic nerve chronic constriction injury (CCI) in female and male rats. Our results evidence excessive, uninhibited proteolytic MMP-9 activity in late-phase (day 28) post-CCI and suggest that the roles of MMP-9 in peripheral neuropathy are universal in both sexes.

## Results

In the peripheral nerve, MMP-9 is a pro-inflammatory protease essential in the initial immune response to injury [6–16, 19]. Recent work has shown that acute/innate and late-phase/adaptive immune response to nerve trauma differentially contributes to the development of neuropathic pain in male and female rodents, respectively [2]. In the present study, utilizing a well-established model of sciatic nerve CCI mononeuropathy in male and female rats, we tested whether MMP-9 contributes to late-phase painful peripheral nerve trauma or whether the changes in MMP-9 expression, proteolytic activity, or excretion post-CCI are sexually dimorphic.

### Time course of MMP-9 and TIMP-1 expression in sciatic nerve post-CCI

**Fig. 1:**
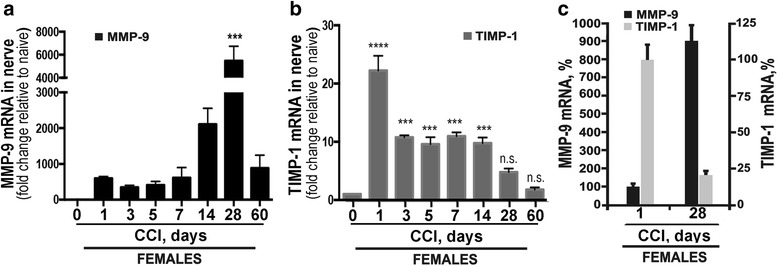
Late-phase MMP-9, but not TIMP-1, mRNA upregulation in the nerve post-CCI. Taqman qRT-PCR for MMP-9 (**a**) and TIMP-1 (**b**) in female rat sciatic nerve at day 0 (naïve) and days 1, 3, 5, 7, 14, 28, and 60 post-CCI. The mean relative mRNA ± SEM of *n* = 4/group was normalized to GAPDH, calibrated to the naïve nerve, and analyzed by one-way ANOVA and Tukey’s post hoc test (****p < 0.0001; ***p < 0.001; n.s., non-significant, compared to the naïve nerve). **c** The fold difference in the MMP-9 (black) and TIMP-1 (gray) mRNA levels at day 1 and day 28 post-CCI in (**a**, **b**). Expressed as a percentage relative to the respective value at day 1 (= 100%)

There is a consensus that the MMP-9:TIMP-1 ratio largely determines the net proteolytic activity of the MMP-9 enzyme. We used our established Taqman qRT-PCR methods [7, 10, 12, 18] to quantify the relative MMP-9 and TIMP-1 expression levels and MMP-9:TIMP-1 expression ratio in the sciatic nerve between day 0 and 60 post-CCI in female rats (Fig. 1). As females are prevalent sufferers of chronic pain [20, 21], historically [7–13, 22, 23] and initially in the present study, we used female rats. In the naïve sciatic nerve (day 0), MMP-9 mRNA level was exceedingly low, whereas the TIMP-1 mRNA level was high (Fig. 1a). After CCI injury, MMP-9 mRNA level at the nerve injury site increased within 1 day and persisted for several weeks, escalating again at day 28 post-CCI. Relative to the naïve nerve, TIMP-1 mRNA peaked at day 1 post-CCI but then declined over the time course (Fig. 1b). At day 28 post-CCI, the levels of TIMP-1 and MMP-9 were reduced ~ 5-fold and increased ~ 9-fold relative to day 1, respectively (Fig. 1c). These findings suggested that in the injured nerve microenvironment, the MMP-9:TIMP-1 ratio likely shifted in favor of MMP-9 enzyme at day 28 post-CCI relative to day 1; although due to the high basal level of the TIMP-1 expression in adult rat nerve [7, 17], the presence of TIMP-1-free MMP-9 activity at day 28 post-CCI cannot be assumed. We next aimed to confirm the presence of active MMP-9 at day 28 post-CCI.

### Late-phase proteolytic MMP-9 activity in female and male CCI nerve

**Fig. 2:**
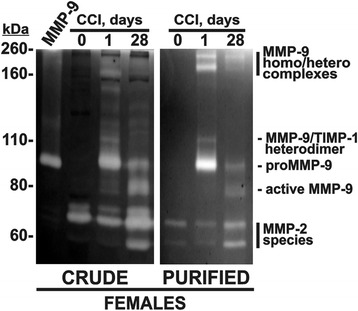
Late-phase catalytic MMP-9 activity in the nerve post-CCI. Gelatin zymography of the crude sciatic nerve extract (10 μg each; CRUDE) and the purified MMP-9 samples (from 25 μg total extract each; PURIFIED) isolated at day 0 (contralateral to injury, at day 1) and days 1 and 28 post-CCI in female rats (representative of *n* = 4/group). MMP-9 the latent MMP-9 control from HT1080 cells

To confirm the presence of active MMP-9 at late-phase nerve injury, we performed gelatin zymography of crude nerve extracts at two critical time points in which the MMP-9 mRNA level increased (days 1 and 28 post-CCI) in female rat nerves (Fig. 2). Contralateral female rat nerve at day 1 post-CCI served as control. In addition, prior to analysis by gelatin zymography, we purified MMP-9 from the nerve extracts using gelatin-Sepharose beads that capture MMP-9 via its gelatin-binding site, a repeat of three fibronectin type-II modules inserted in the catalytic domain [24]. In both the crude and purified samples, MMP-9 was not detected in the normal nerve (day 0), whereas MMP-9 was dramatically upregulated in the injured nerve at day 1 post-CCI (Fig. 2). The gelatinolytic activity bands in the injured nerve samples corresponded to the known species of MMP-9 that included the 92-kDa proenzyme, the 84-kDa active mature enzyme, the 120–125-kDa MMP-9●TIMP-1 complex, and the multiple 200–260-kDa MMP-9 homo/heterodimers [4, 7, 25] and was consistent with our prior observations [7, 8]. A significant increase in the 84-kDa active, mature MMP-9 enzyme was particularly evident in the nerve samples at day 28 post-CCI in female rats. Both the MMP-2 proenzyme and the active enzyme were detectable in normal and, at higher levels, injured CCI nerves, consistent with prior observations [6–16].

**Fig. 3:**
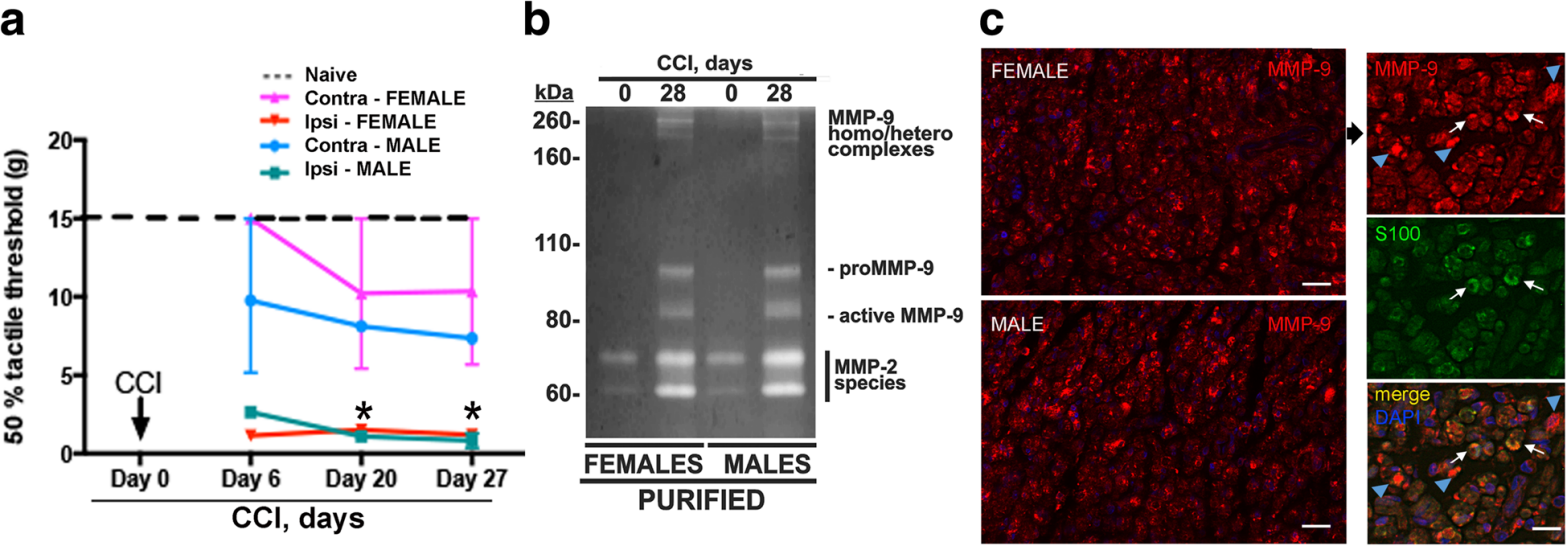
Late-phase MMP-9 in female and male nerves post-CCI. **a** von Frey testing in female and male rat CCI ipsilateral and contralateral to injury in hind paws. The graph represents the mean withdrawal thresholds (gram force, g) ± SEM, displaying significant allodynia in both female and male animals at day 28 post-CCI in the ipsilateral hind paw (*p ≤ 0.05). **b** The status of catalytic MMP-9 activity in the sciatic nerve in female and male rats. Gelatin zymography of the purified MMP-9 samples (from 25 μg total extract each; PURIFIED) isolated at day 28 post-CCI contralateral (day 0) and ipsilateral (day 28 post-CCI) to injury (representative of *n* = 4/group). **c** Left panel: MMP-9 immunostaining (red) in the sciatic nerve in female and male rats at 4 weeks post-CCI (representative of *n* = 3/group). Scale bar, 50 μm. Right panel: MMP-9 (red) and S100 (green) dual immunostaining in female nerve demonstrates that Schwann cells (white arrows) and other cells (blue arrowheads) produce MMP-9 at day 28 post-CCI. Scale bar, 25 μm

Because the late-phase, adaptive immune response to painful nerve injury differs in female and male rodents [2], we comparatively assessed sensitivity to pain using von Frey testing and then the late-phase MMP-9 activity in nerves using gelatin zymography in female and male rats at day 28 post-CCI. Both female and male rats displayed sustained pain hypersensitivity at week 4 post-CCI (Fig. 3a), consistent with the findings of others [26–29]. Relative to the normal contralateral to injury (day 0) nerves, MMP-9 at the nerve injury site (day 28) was upregulated and activated similarly in female and male samples (Fig. 3b). No significant difference was observed in the intensity and the species of MMP-9 or MMP-2 bands between the female and male nerves (Fig. 3b). There was also no apparent difference seen in the distribution of MMP-9 immunoreactivity at day 28 post-CCI between the female and male nerves (Fig. 3c). In both groups, MMP-9 reactivity was observed in various cells, including crescent Schwann cells, as confirmed by dual-immunofluorescence for MMP-9 and Schwann cell marker, S100 (Fig. 3c).

Together, these data confirm the earlier observation of the injury-specific MMP-9 upregulation in Schwann cells and other cells of peripheral nerve [6–16] and provide the first evidence for a drastic increase in MMP-9 expression and catalytic activity in the late-phase painful neuropathy, which is comparable in female and male rodents.

### Post-nerve injury MMP excretion via the urine in female and male rats

**Fig. 4:**
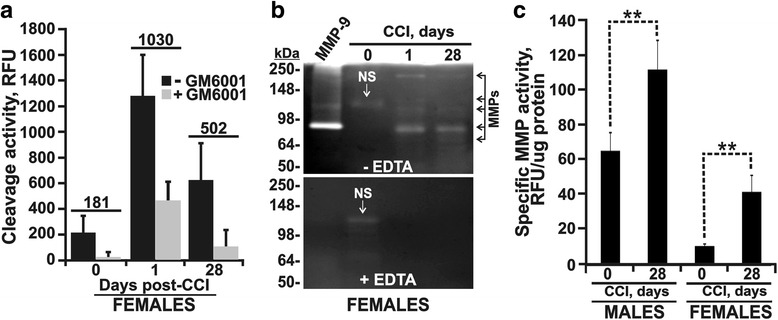
MMP excretion in female and male rats post-CCI. **a** MMP activity in urine of female rats. The urine samples collected at day 0 (prior to injury) and days 1 and 28 post-CCI (*n* = 4/group) were equilibrated in MMP buffer, pH 7.5, and then the protein concentrations were determined using the Bradford assay and made even by sample dilution in MMP buffer, pH 7.5. Dialyzed urine (50 μl each) was co-incubated with the fluorescent Mca-PLGL-Dpa-AR-NH_2_ MMP substrate. Where indicated, GM6001 (10 μM) was added to the samples to inhibit MMP activity. The numbers indicate the MMP-specific cleavage activity. Data are means ± SD from multiple individual measurements performed at least in duplicate. **b** Gelatin zymography of female rat urine (5 μl of each normalized sample) collected at day 0 and days 1 and 28 post-CCI (representative of *n* = 4/group). Gels were incubated in the absence and the presence of 20 mM EDTA (− EDTA and + EDTA, respectively). **c** Specific urinary MMP activity in female and male rats. The urine samples collected at day 0 and 28 post-CCI (*n* = 4–5/group) were equilibrated in MMP buffer, pH 7.5, and then the protein concentrations were determined using the Bradford assay. Dialyzed urine samples (50 μl, each) were co-incubated with the fluorescent Mca-PLGL-Dpa-AR-NH_2_ MMP substrate in the presence and the absence of GM6001 (10 μM). The specific MMP activity (RFU without GM6001–RFU with GM6001) was normalized to the protein concentrations. Data are means ± SD from multiple individual measurements performed at least in duplicate. RFU relative fluorescence unit, MMP-9 the latent MMP-9 control from HT1080 cells, NS non-MMP activity band

Glycoproteins, including MMP-9, are normally excreted via the urine [30, 31]. Protease levels are increased in the urinary proteome of painful (diabetic) neuropathy patients [32]. MMP-9 is a glycoprotein with multiple *O*-glycosylation sites in the linker region between the catalytic and hemopexin domains, and two *N*-glycosylation sites in the inhibitory prodomain [33–35]. To investigate if the observed increase in MMPs in the injured nerve was followed by an elevated MMP excretion in the urine and if the urinary MMP excretion was distinct in males and females, we employed two independent methods to assess protolytic MMP activity in the rat urine samples: (1) the total MMP activity using the Mca-PLGL-Dpa-AR-NH_2_ fluorescent peptide as a broad-specificity MMP cleavage substrate (Fig. 4a) and (2) gelatin zymography (Fig. 4b) analyses.

To estimate the total MMP activity in the urine samples before and after CCI, we first used the Mca-PLGL-Dpa-AR-NH_2_ fluorescent peptide as a broad-specificity MMP cleavage substrate (Fig. 4a). To limit interference of the impurities in the crude samples with the fluorogenic substrate assay, we dialyzed the urine against the MMP buffer, pH 7.5, prior to the assay. To determine the specific MMP cleavage of the substrate, the dialyzed samples were incubated with the Mca-PLGL-Dpa-AR-NH_2_ substrate in the presence and absence of GM6001, a broad-range hydroxamate inhibitor of MMPs. In female rats, there was a significant, roughly 5–6-fold increase in the total MMP activity in the day 1 urine samples post-CCI relative to day 0. The elevated urinary MMP activity, still by 3-fold relative to prior nerve injury, sustained at least until day 28 post-CCI. Since GM6001 greatly repressed the cleavage activity in the urine samples, we concluded that non-MMP proteases did not significantly contribute to the substrate cleavage.

To support these findings, we examined the status of the gelatinolytic MMPs in the rat urine samples using gelatin zymography (Fig. 4b). The level of the urinary MMP activity in the sample at day 0 was below detection limit, except for one gelatinolytic band. Relative to day 0, there was a significant increase in the urinary MMPs at day 1 post-CCI that persisted until day 28 post-CCI. To confirm the MMP identity of the gelatinolytic bands, we used EDTA, a metal chelator and a general inhibitor of zinc-MMPs. EDTA repressed all of the MMP-related gelatinolytic bands in the samples, except a single EDTA-resistant band in the day 0 samples, suggesting the presence of minor non-MMP gelatinolytic activity in the normal rat urine.

To compare the MMP excretion patterns in female versus male rats post-CCI, we quantified the MMP-specific activity levels in the urine before (day 0) and at day 28 post-CCI in the same animal cohort (Fig. 4c). The measurements demonstrated that, although males exhibited an elevated constitutive urinary MMP activity compared with females, the change in MMP activity at day 28 post-CCI relative to the respective pre-injury control increased similarly in males and females (~ 40–50 RFU/μg total protein). Overall, an enhanced MMP excretion in the urine corroborates the MMP upregulation in the injured nerve that is universal in both sexes.

## Discussion

Despite the high regenerative capacity of the peripheral nerve, nerve trauma often results in poor functional recovery and debilitating neuropathic pain [36–38]. Chronic neuroinflammation is a major contributing factor in the development of neuropathic pain [39–41]. Chronic constriction injury (CCI) to rat sciatic nerve is an animal model for the study of the mechanisms underlying clinically relevant pain-like behaviors from normally painless stimuli (allodynia) and exaggerated pain from painful stimuli (hyperalgesia) sustained for weeks after incitement of CCI [26–29]. In the models of painful peripheral neuropathy, the MMP enzyme family has multiple established and critical roles in immune cell recruitment and demyelination and the development of neuropathic pain [6–16, 19, 39, 42].

MMP-9 is a unique early-response proinflammatory MMP family member induced in adult nerve exclusively after injury, including CCI [6, 8, 10–12, 14, 23, 43–45], as confirmed in the present study. Proinflammatory cytokines, including TNF-α and IL-1β, stimulate MMP-9 expression in Schwann cells and endothelial cells of the nerve [11, 12]. Infiltrating immune cells, such as neutrophils and macrophages, serve as additional sources of MMP-9 in the damaged nerve. A number of the early-phase MMP-9 activities in nerve injury have been established, including immune cell infiltration, suppression of Schwann cell mitosis, and promoting demyelination by degradation of myelin protein [7–12, 44]. In addition to tissue remodeling at the nerve injury site, MMP-9 is thought to contribute to pro-nociceptive dorsal spinal cord plasticity after peripheral nerve injury [6, 46, 47]. In support of the net proinflammatory early-phase gelatinolytic activity in painful neuropathy, systemic acute MMP-9/2 inhibition at day 1 post-CCI prevents the development of neuropathic pain [8]. In addition, broad-spectrum MMP inhibition [44] and MMP-9 targeting using siRNA [19] protect from nerve injury-induced pain.

The present study offers the first evidence that MMP-9 expression and activity are elevated in the nerve during late-phase nerve injury. Because of a multi-fold shift of the MMP-9:TIMP-1 ratio in favor of MMP-9, the excess MMP-9 activity was unencumbered by its endogenous inhibitor TIMP-1 specifically during the late-phase. These data are in contrast to the findings of the early-phase MMP-9 increase as inactive pro-enzyme and add significantly to the previous studies implicating the early-phase action of MMP-9 in pain [8, 19, 44]. In addition to pain, the early-phase Schwann cell anti-mitogenic MMP-9 action during the first days post-nerve crush affects long-term nerve repair in part by regulating Schwann cell numbers in the regenerating nerve [7, 9, 10]. Using MMP-9 knockout animals, we have established the essential role of MMP-9 in voltage-gated sodium (Nav) channel clustering in the nodes of Ranvier, and MMP-9 deletion resulted in excessively large Nav channel clusters at 1 month post-crush [7]. This finding suggests that by regulating remyelination, MMP-9 may contribute to pain resolution. Insofar, the direct pro-regenerative role of gelatinases in the sciatic nerve has been attributed to MMP-2, and not MMP-9, via degradation of growth-inhibitory proteoglycans [48, 49].

The peripheral nerve comprises a plethora of MMP-9 substrates. By regulation of the cleavage and the release of soluble cytokine and trophic factor ligand and receptors from their membrane isoforms, MMP-9 activity regulates activation and inactivation of cytokine and trophic pathway in the damaged nerve [10, 39, 43]. By proteolytic fragmentation of myelin basic protein (MBP) and myelin-associated glycoprotein (MAG) of nerves, MMP-9 activity promotes demyelination and the release of the pro-algesic MBP [8, 22, 50] and growth-inhibitory MAG [51] fragments, respectively. MMP-9 controls Nav channel clustering in the injured nerve by proteolysis of laminin and dystroglycan, both required for Nav clustering [7, 52], or by direct proteolysis of the extracellular loop region of Nav channels [53]. The evidence of the active MMP-9 species in the nerve at the late-phase painful neuropathy offers a possibility for MMP-9 role in pain sustenance or resolution via control of nerve regeneration, demyelination, and ion channel functioning. Our studies also indicate multiple roles of catalytic MMP-9 (defined by inhibition using GM6001 inhibition) in Schwann cell signaling; it produced a bi-phasic ERK1/2 activation by proteolytic activation of insulin-like growth factor 1, epidermal growth factor, and platelet-derived growth factor signaling pathways [10]. In turn, by inhibiting excess MMP-9 proteolysis, TIMP-1 is expected to promote nerve regeneration and remyelination [7, 18] and to inhibit the development of neuropathic pain [19, 54]. Our data suggest that insufficient TIMP-1 level to limit late-phase MMP proteolysis in the damaged nerve provides rationale for the use of late-phase TIMP-1-based therapy in painful neuropathy.

In addition to proteolysis of extracellular proteins and protein domains [55, 56], MMP-9 regulates cell behavior by direct receptor binding [25, 57–59]. The unique representation of the multiple MMP-9 isoforms, including the proenzyme, mature enzyme, homodimers, and heterodimers, in the injured nerve suggests its complex and multiple roles. At day 1 post-CCI, high levels of inactive MMP-9 proenzyme are consistent with high TIMP-1 level. In excess of latent MMP-9 over TIMP-1, MMP-9 proenzyme free from TIMP-1 is activated by plasmin or MMP-3 [60]. In addition, MMP-9 proenzyme may be involved in signaling neurite growth or Schwann cell migration through interaction of the non-catalytic hemopexin domain with low-density lipoprotein receptor-related protein-1 [61, 62] or through the cell surface complex with CD44, mediating TIMP-1-induced survival signaling [63]. Thus, the spatial and temporal relation of the specific MMP-9 and TIMP-1 species and binding partners will determine the functional outcomes of their individual and joint activities in painful peripheral neuropathy.

MMP-9 is a glycoprotein with multiple *O*-glycosylation sites in the linker region between the catalytic and hemopexin domains and, in addition, two well-defined *N*-glycosylation sites—one in the prodomain and another in the catalytic domain [33–35]. A majority of the secretory and cell surface proteins that are shed into the urine are glycosylated [30, 64]. The urinary MMPs are elevated in multiple cancer and nephropathy types, osteoarthritis, and heart failure [31, 65–68] and constitutively in male compared to female rat urine. Similarly, we recorded a significant increase in the total MMP cleavage activity as well as the presence of the elevated levels of the multiple MMP species in rat urine post-nerve injury. While the non-invasive experimental urine measurements readily distinguished animals with painful neuropathy from normal animals, the presence of non-neuropathic conditions with elevated urinary MMPs [31, 65–68] cannot be excluded in the clinical setting. Nevertheless, characterization of urinary extracellular vesicles present considerable research interest due to their possible use as a source of biomarkers and have been shown to contain proteases in patients with diabetic neuropathy [32].

Understanding the influence of biological variables, including subject’s sex, on pathophysiology of painful neuropathy is critical to the development of personalized therapeutic and diagnostic approaches. Because females are more common sufferers of chronic pain [20, 21], our historical [7–13, 23] and initial experiments in the present study employed female rat CCI. Comparative studies of immune response to nerve injury in female and male rodents indicated clearly that immunotherapeutic targeting of mechanical allodynia is sex-dependent [2]. Because MMP-9 expression, activity, and excretion are universal in female and male rats with painful neuropathy, in the search for selective markers of nerve injury and neuropathic pain, MMP-9 presents a reliable, injury-specific surrogate biomarker regardless of subject’s sex.

## Conclusion

In conclusion, the present study offers the first evidence for the excessive, uninhibited proteolytic MMP-9 activity during late-phase painful peripheral neuropathy and suggests that the pattern of MMP-9 expression, activity, and excretion after peripheral nerve injury is universal in both sexes. Future studies identifying detrimental and beneficial roles of MMP-9 in pain resolution and nerve repair in late-phase nerve injury are warranted.

## Methods

### Reagents

All reagents were purchased from MilliporeSigma unless indicated otherwise. The horseradish peroxidase (HRP)-conjugated goat anti-rat IgM (#3020-05) was purchased from Southern Biotech. The HRP-conjugated goat anti-rat IgG (#112-035-175) and the [(7-methoxycoumarin-4-yl)acetyl]-Pro-Leu-Gly-Leu-[N-3-(2,4-dinitrophenyl)-L-2,3-diamino-propionyl]-Ala-Arg-NH_2_ (Mca-PLGL-Dpa-AR-NH_2_) fluorescent MMP substrate were obtained from Jackson ImmunoResearch and R&D Systems, respectively. A 3,3′,5,5′-tetramethylbenzidine substrate (TMB/E) and protease-free BSA (a 30% solution) were from Surmodics and US Biological, respectively. Gelatin-Sepharose 4B beads and Micro Bio-Spin columns were from GE Healthcare and Bio-Rad, respectively. The Coomassie protein assay reagent and Novex 10% zymogram (0.1% gelatin) gels were purchased from Thermo Scientific.

### Animal model and sample collection

Sixty adult female and 20 male Sprague-Dawley rats (200–225 g) were obtained from Envigo Labs and housed in a temperature-controlled room (22 °C) on a 12-h light/dark cycle with free access to food and water. Following anesthesia with 4% isoflurane in oxygen (Aerrane; Baxter), the common sciatic nerve was exposed unilaterally at the mid-thigh level. The nerve received three loosely constrictive chromic gut ligatures to produce CCI [26]. At days 0–60 post-CCI, urine sample aliquots (0.2–0.4 ml) were collected, readily placed on ice for a few minutes, and then cleared by centrifugation (2,000×*g*; 10 min; 4 °C). Cleared urine aliquots were equilibrated in 50 mM HEPES, pH 7.5, containing 10 mM CaCl_2_, 0.5 mM MgCl_2_, and 10 μM ZnCl_2_ (MMP buffer, pH 7.5), using a desalting spin column and immediately used in the MMP activity tests. Sciatic nerve samples were collected and snap-frozen in liquid N_2_ and stored at − 80 °C for protease activity assays and in RNA-later and stored at − 20 °C for the qRT-PCR analyses. For immunohistochemistry, sciatic nerves were isolated following transcardial perfusion in 4% paraformaldehyde in 0.2 M phosphate, post-fixed and embedded in paraffin. Animals were sacrificed using Beuthanasia (150 mg/ml; i.p., Schering-Plough Animal Health). All animal procedures were performed according to the PHS Policy on Humane Care and Use of Laboratory Animals and the protocol approved by the Institutional Animal Care and Use Committee at the VA San Diego Healthcare System.

### von Frey testing

Rats were habituated to the testing environment prior to baseline tests. Testing was performed daily for 3 consecutive days prior to and then at the indicated time points after CCI. Rats were placed in individual Plexiglas compartments with wire mesh bottom, and von Frey filaments (0.41–15.2 g, Stoelting) were applied perpendicularly to the mid hind paw and held for 4–6 s. A positive response was noted if the paw was sharply withdrawn. The 50% probability of withdrawal threshold was determined by Dixon’s up-down method [69].

### Taqman qRT-PCR

Taqman primers and a probe containing 5′-FAM reporter for rat TIMP-1 (GenBank, NM_053819) were from Applied Biosystems (cat. # Rn01430873_g1). Primers and probes for MMP-9 (GenBank, NM_031055) and glyceraldehyde 3-phosphate dehydrogenase (GAPDH; GenBank, X02231) were from Biosearch Technologies [8, 12]. Total RNA was extracted using TRIzol and purified on an RNeasy mini column (Qiagen). The RNA purity was estimated by measuring the A_260/280_ and the A_260/230_ ratios. The samples were treated with RNase-free DNAse I (Qiagen). cDNA was synthesized using a first-strand cDNA kit (Roche). Gene expression levels were measured in a Mx3005P (Agilent) using 50 ng cDNA and 2×Taqman Universal PCR Master Mix (Applied Biosystems) with a one-step program: 95 °C, 10 min; 95 °C, 30 s; 60 °C, 1 min for 50 cycles. Using the injured sciatic nerve cDNA samples, primers (Biosearch Technologies) and Taqman probes for MMP-9 (Roche) and TIMP-1 (Applied Biosystems) were optimized to reach the amplification efficiency of 100.1–100.3% [12]. GAPDH was used as a normalizer; its expression changes were insignificant in the injured relative to naïve nerves. Samples without cDNA (a no template control) showed no contamination. Relative mRNA levels were quantified using the comparative delta Ct method [70]. The fold change between CCI and naïve samples was determined using the Mx3005P software.

### Protease activity assay

The cleavage assay was performed in a 0.2-ml total volume in wells of a 96-well plate (Thermo Scientific) using the fluorescent Mca-PLGL-Dpa-AR-NH_2_ peptide substrate (1 μM) and the 50-μl urine aliquots equilibrated in the MMP buffer, pH 7.5. Where indicated, GM6001 (10 μM) was co-incubated for 30 min at ambient temperature with the urine samples to inactivate MMPs. Initial reaction velocity was monitored continuously at *λ*_ex_ = 320 nm and *λ*_em_ = 400 nm using a fluorescence spectrophotometer. Data are means ± SD from several independent experiments performed at least in duplicate. Protein concentrations in the dialyzed urine samples were determined using the Coomassie protein assay.

### MMP-9 purification using gelatin-Sepharose beads

The proteins were extracted for 1 h at 4 °C from the sciatic nerve samples using 50 mM Tris-HCl buffer, pH 7.4, containing 150 mM NaCl, 1% Triton X-100, 0.1% SDS, 10 mM EDTA, the protease cocktail inhibitor, and 1 mM phenylmethylsulfonyl fluoride. The protein concentration of the extracts was determined using the Coomassie protein assay and adjusted to reach 1 mg/ml. The extract aliquots (100 μg total proteins, each) were 10-fold diluted using the above buffer lacking Triton X-100 and SDS and allowed to bind to gelatin-Sepharose 4B beads for 16–18 h at 4 °C. Following extensive washing, the bound material was eluted using non-reducing 2×SDS sample buffer (50 μl).

### Gelatin zymography

The dialyzed rat urine samples equilibrated in MMP buffer, pH 7.5; the crude nerve extracts; and the MMP-9 samples isolated from sciatic nerve were analyzed by gelatin zymography in a 10% acrylamide-0.1% gelatin gel. Gels were next processed as described previously to visualize the clear gelatinolytic activity bands [7, 8]. Where indicated, gels were incubated in 20 mM EDTA to inactivate MMP activity.

### Immunohistochemistry

Transverse (10-μm-thick) sciatic nerve sections collected at 4 weeks post-CCI after transcardial perfusion in 4% paraformaldehyde in 0.2 M phosphate, post-fixed and embedded in paraffin, were deparaffinized in xylenes and rehydrated in graded ethanol. Following endogenous aldehyde group block (0.5% sodium borohydride in 1% sodium dibasic buffer for 5 min) and non-specific binding block (5% goat serum in PBS for 30 min at room temperature), nerve sections were incubated with a rabbit polyclonal antibody to MMP-9 (1:500; Torrey Pines Biolabs, 16–18 h at 4 °C) alone or with rabbit polyclonal antibody to MMP-9 (1:50; SantaCruz, 16–18 h at 4 °C) sequentially with mouse monoclonal antibody to S100 (1:2000, Sigma, 16–18 h at 4 °C), followed by the respective species-specific Alexa 594 (red) or Alexa 488 (green)-conjugated secondary antibody (ThermoFisher Scientific, 1 h, room temperature, each). PBS was used for rinsing. Slowfade Gold antifade reagent containing DAPI (4′,6-diamidino-2-phenylindole, ThermoFisher Scientific, blue) was used for mounting. Staining specificity was confirmed by a primary antibody omission. The images were acquired using All-In-One Fluorescence Microscope BZ-X700 (Keyence, Itasca, IL).

### Statistical analyses

Statistical analyses were performed using Graph Prism 6 (Synergy Software) or SPSS 16.0 (SPSS) software by a two-tailed, unpaired Student’s *t* test or one-way analyses of variance (ANOVA) with multiple comparisons followed by Tukey’s post hoc test. *p* ≤ 0.05 values were considered significant.

## Abbreviations

CCI: Chronic constriction injury
HRP: Horseradish peroxidase
Mca-PLGL-Dpa-AR-NH_2_: [(7-methoxycoumarin-4-yl) acetyl]-Pro-Leu-Gly-Leu-[N-3-(2,4-dinitrophenyl)-L-2,3-diamino-propionyl]-Ala-Arg-NH_2_
MMP buffer: 50 mM HEPES, pH 7.5, containing 10 mM CaCl_2_, 0.5 mM MgCl_2_, and 10 μM ZnCl_2_
MMP: Matrix metalloproteinase
TIMP: Tissue inhibitor of MMP
